# Supplementary material for: Practices and Barriers in Implementing the Low FODMAP Diet for Irritable Bowel Syndrome Among Malaysian Dietitians: A Qualitative Study
Source: Nutrients. 2024 Oct 23;16(21):3596. doi: 10.3390/nu16213596 (PMC11547897; doi:10.3390/nu16213596)
Supplement: Supplementary file 1 [file nutrients-16-03596-s001.zip › nutrients-3186519-supplementary.pdf]

Table S1. Interview guideline.

Themes	Main Questions
**Introduction**	1. Can you tell me a little bit of your background?<br>2. How often do you receive referral for IBS patients from doctors?<br>3. Do you generally recommend the low FODMAP diet to all IBS patients?
**Practice**	1. Can you share with me what you know about the low FODMAP diet?<br>2. Can you share with me your understanding about the restriction phase? How do you assist your patients in identifying foods/beverages high in FODMAPs? (resources)<br>3. How about reintroducing FODMAPs back into the diet?
**Barriers**	1. What are the difficulties you face in implementing the diet?<br>2. How do you overcome the barriers?
